# The role of active movement in fungal ecology and community assembly

**DOI:** 10.1186/s40462-019-0180-6

**Published:** 2019-11-20

**Authors:** Miloš Bielčik, Carlos A. Aguilar-Trigueros, Milica Lakovic, Florian Jeltsch, Matthias C. Rillig

**Affiliations:** 1 Institut für Biologie, Plant Ecology, Freie Universität Berlin, 14195 Berlin, Germany; 2 Berlin-Brandenburg Institute of Advanced Biodiversity Research (BBIB), 14195 Berlin, Germany; 3 Department of Plant Ecology and Nature Conservation, University of Potsdam, Am Mühlenberg 3, 14476 Potsdam-Golm, Germany

**Keywords:** Filamentous fungi, Microbial community, Active movement, Modular organisms, Interference competition, Fungal space searching algorithms, Fungal foraging, Fungal highways, Clonal plants, Slime molds

## Abstract

Movement ecology aims to provide common terminology and an integrative framework of movement research across all groups of organisms. Yet such work has focused on unitary organisms so far, and thus the important group of filamentous fungi has not been considered in this context. With the exception of spore dispersal, movement in filamentous fungi has not been integrated into the movement ecology field. At the same time, the field of fungal ecology has been advancing research on topics like *informed growth*, *mycelial translocations*, or *fungal highways* using its own terminology and frameworks, overlooking the theoretical developments within movement ecology. We provide a conceptual and terminological framework for interdisciplinary collaboration between these two disciplines, and show how both can benefit from closer links: We show how placing the knowledge from fungal biology and ecology into the framework of movement ecology can inspire both theoretical and empirical developments, eventually leading towards a better understanding of fungal ecology and community assembly. Conversely, by a greater focus on movement specificities of filamentous fungi, movement ecology stands to benefit from the challenge to evolve its concepts and terminology towards even greater universality. We show how our concept can be applied for other modular organisms (such as clonal plants and slime molds), and how this can lead towards comparative studies with the relationship between organismal movement and ecosystems in the focus.

## Introduction

With their role in organic matter decomposition and plant symbiosis, filamentous fungi are of tremendous importance in all terrestrial ecosystems. They are important human, plant, and animal pathogens, and they are widely used in biotechnological and food industry. Despite this importance, the research on their community assembly has been lagging behind the more easily assayed communities of plants and animals. However, due to methodological developments in microbiology, we have now opportunities to expand our knowledge on fungal community assembly [1]. We advocate that explicitly recognizing the role of fungal *active movement* will help us with this task.

In living organisms, movement occurs in a myriad of ways and on all levels of organization. This has been traditionally reflected in the division of life sciences into disciplines: cellular biology considers cytoplasmic flow or movement of organelles; physiology studies blood flow; while developmental biology describes changes in body part positions during growth. Movement ecology focuses on the movement of entire organisms and their propagules within the environment when searching for food, suitable habitats, reproduction, or avoiding danger.

However, this traditional distinction of biological movement into different domains of life sciences applies only for motile unitary organisms and propagules of sessile organisms. It becomes problematic once we consider modular organisms such as filamentous fungi. Their bodies are composed of filaments (hyphae) interconnected into a mycelial network. In the form of this network, hyphae forage by growing into new areas, and resource patches are integrated through cytoplasmic transport [2]. Fungi use these very specific movement means to respond to the universal challenges presented by a heterogeneous environment. In filamentous fungi, the physiological, developmental and ecological functions of movement are not present as distinctive physical phenomena. They are intertwined within the dynamic processes of a filamentous body, and this often leads to the ecological function of movement being rather overlooked by researchers.

We argue that both mycology and movement ecology can benefit from the explicit recognition of what we refer to as *active moment* in filamentous fungi: the translocation of biomass within the environment brought about by the organism’s own energy resources. Using this term, we proceed to introduce fungi into the movement ecology framework in two steps, which correspond to the conceptual developments within the field of movement ecology itself: In the first step, we draw upon the original concept by Nathan et al. [3] to demonstrate the presence of navigation and motion capacity in filamentous fungi. We also propose a definition of *active movement* which is: i) inclusive of all groups of organisms, unitary and non-unitary, motile and sessile, ii) and thus also extends the concept of the movement path towards a more diverse array of active biomass translocations. In the second step, we use the extended movement ecology framework by Jeltsch et al. [4] that links movement ecology with biodiversity research to further strengthen our case for the recognition of *active movement* in filamentous fungi by showing that just like in other groups, the movement of a filamentous fungus has an effect on (microbial) community composition both via mobile linkers (for example, bacteria can use fungi for dispersal), or by acting as a factor of intraspecific and interspecific interactions between fungi.

Thus, we revisit relevant existing research in fungal ecology with the link between movement and species coexistence in mind. Therefore, this paper has two closely related aims: One aim is to use the movement ecology framework to define the *active movement* in filamentous fungi and to provide theoretical background to disentangle the ecological function of hyphal and mycelial movements from physiological and developmental functions. In doing so, we provide a concept that enables movement ecologists to tap into the research in filamentous fungi ecology. Second, we argue that the explicit recognition of *active movement* and adoption of movement ecology terminology will provide a more comprehensive treatment of the ecological implications of movement in fungi, and will fuel a new line of research in fungal ecology and community assembly.

In sum, we think that our work will benefit both fungal and movement ecology. This type of fungal movement (i.e. *active movement* in the mycelial network) has not been recognized within the movement ecology framework as opposed to the relatively better studied dispersal by spores. For movement ecology, describing filamentous fungi using the common terminology of movement ecology opens an opportunity to challenge the universality of its basic frameworks and terminologies. We demonstrate this improvement towards greater universality also by showing how other modular organisms, namely clonal plants and slime molds, can fit into our concept. We provide examples of how our concept can aid the comparative ecological studies between these groups, and the use of microbes as model organisms for movement ecology.

As mentioned earlier, fungal dispersal by spores is not within the scope of this article. However, we want to prevent our concept of *mycelial active movement* from being (mis)interpreted as an antipode to the seemingly *passive* dispersal by spores. First, spores can be actively moved by forces generated by the parental mycelium [5, 6]. Second, we support the broad definition of navigation and motion capacity sensu Nathan [3], which accommodates evolved traits such as *when* or *how many* spores are released as a part of mycelial navigation capacity; as well as traits which make spores stick to mobile linkers (i.e. animal dispersal), or survive longer journeys, as a part of movement capacity [7, 8].

## Active movement: definition

Movement is one of the means by which organisms interact with their environment. It enables them to respond to environmental challenges and to access resources. The first step in the process of adding fungal *active movement* to the movement ecology framework requires a revision of the definition of movement itself. We propose the following definition as inclusive for all organisms that interact with their environment in the ways described by the movement ecology framework of Nathan et al. [3] and Jeltsch et al. [4]:

*Active movement is any translocation of biomass sustained by an organism’s own energy resources, which is steered (navigated) in response to environmental cues and stimuli, or by environmental selection pressures, and can in turn result in a direct effect on the biotic and abiotic environment.*

Based on this definition, we show below how features of fungal morphology and physiology can be described as movement traits, how those traits enable the fungus to respond to its environment, and how these responses affect fungal community assembly. In doing so, we also align the (most important) movement ecology and fungal biology terminology.

### Step 1: active movement in filamentous fungi; Nathan’s movement ecology framework

Just like in motile organisms, in filamentous fungi the environmental cues and stimuli can influence the *internal state* of the filamentous fungus, and steer (*navigation capacity*) the translocation of the biomass (*motion capacity*) [3]. This results in a particular spatial location of the fungal biomass in a particular time (*movement path*) [3].

#### Motion capacity

Three different kinds of translocation in hyphae and mycelium can enable a direct response to the environment, and can be recognized as forms of active movement: Hyphal (mycelial) growth [9, 10], transport within the cytoplasm [11, 12], and migration (retraction) of the entire cytoplasm within a hypha [13].

**Fig. 1:**
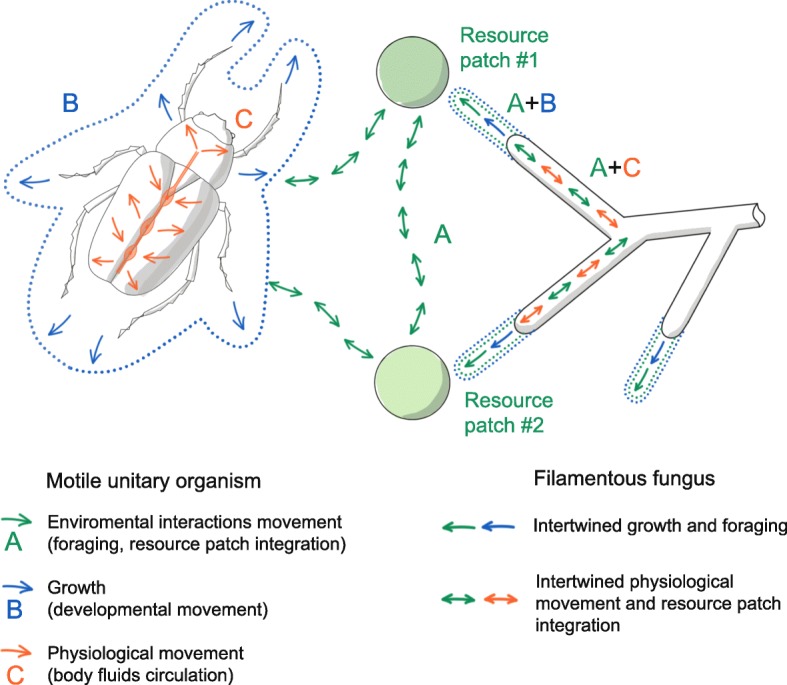
Main movement functions in unitary motile and modular organism (filamentous fungus). In motile unitary organism (left), the individual interacts with the environment by moving its entire body from one point to another (green arrows; **a**). Physiological movements (orange arrows; **c**) and developmental movement (i.e. growth and morphology, blue arrows and dots; **b**) are present as distinctive processes. A filamentous fungus (right) has no capacity to move its entire body. Instead it intertwines the foraging with growth and morphology (green + blue arrows and dots; A + B), and resource patch integration with physiological movements (green arrows + orange arrows; A + C)

In motile unitary organisms, the following is realized as three distinct, decoupled processes (Fig. 1):

(A) translocation of the entire organism (engaging with the heterogeneous environment, resource integration from different locations, escaping or attacking), studied by movement ecology.
(B) growth studied by developmental biology, and
(C) movement of the physiological fluids, maintaining homeostasis.

In contrast, filamentous fungi must respond to the challenges of their heterogeneous environment, homeostasis maintenance and developmental growth by intertwining all (A + B + C): Translocation of the organism is intertwined with growth (A + B). Movement of the physiological fluids can have both a homeostatic function, as well as the function of integrating resources from different patches in the environment (A + C). Also the entire cytoplasm can be moved from one location to another along hyphae (called the “hyphal channel” in fungal biology), and it is worth mentioning that in fungi several forms of cellular death can be seen as movement traits. If the mycelium at older locations degenerates (possibly recycling some of its own biomass) while outgrowing to new locations, the summary result can be a change in position of the entire organism, which is very similar to situations in typical motile organisms. Therefore, also processes such as autophagy should be recognized as movement related traits [14].

We point out that just like in other actively moving groups, motion capacity in fungi differs radically between species. For example, Olsson [15] let different species grow in Petri dishes with a source of concentrated C on one end, and a source of concentrated N on the opposite end, with the gradient of concentrations in between. While some species were able to actively integrate resources across all space and grow in the entire Petri dish, others were only growing in the central part.

In our concept, it is pivotal to make a clear distinction between the two main forms of ecologically relevant movements (movement capacities) in filamentous fungi, i.e. the translocation by informed growth, and the translocation by cytoplasmic transports (Fig. 1). For example, the growth of hyphae is of primary interest in the dispersal of bacteria in soil environment, while the cytoplasmic transport acts in clonal subsidizing. However, it should be noted that in the development of fungal body, cytoplasmic streaming and hyphal growth are closely interrelated. For more details, we refer to the mycofluidics review by Ropert and Seminara [6].

#### Navigation capacity, internal state and movement path

**Fig. 2:**
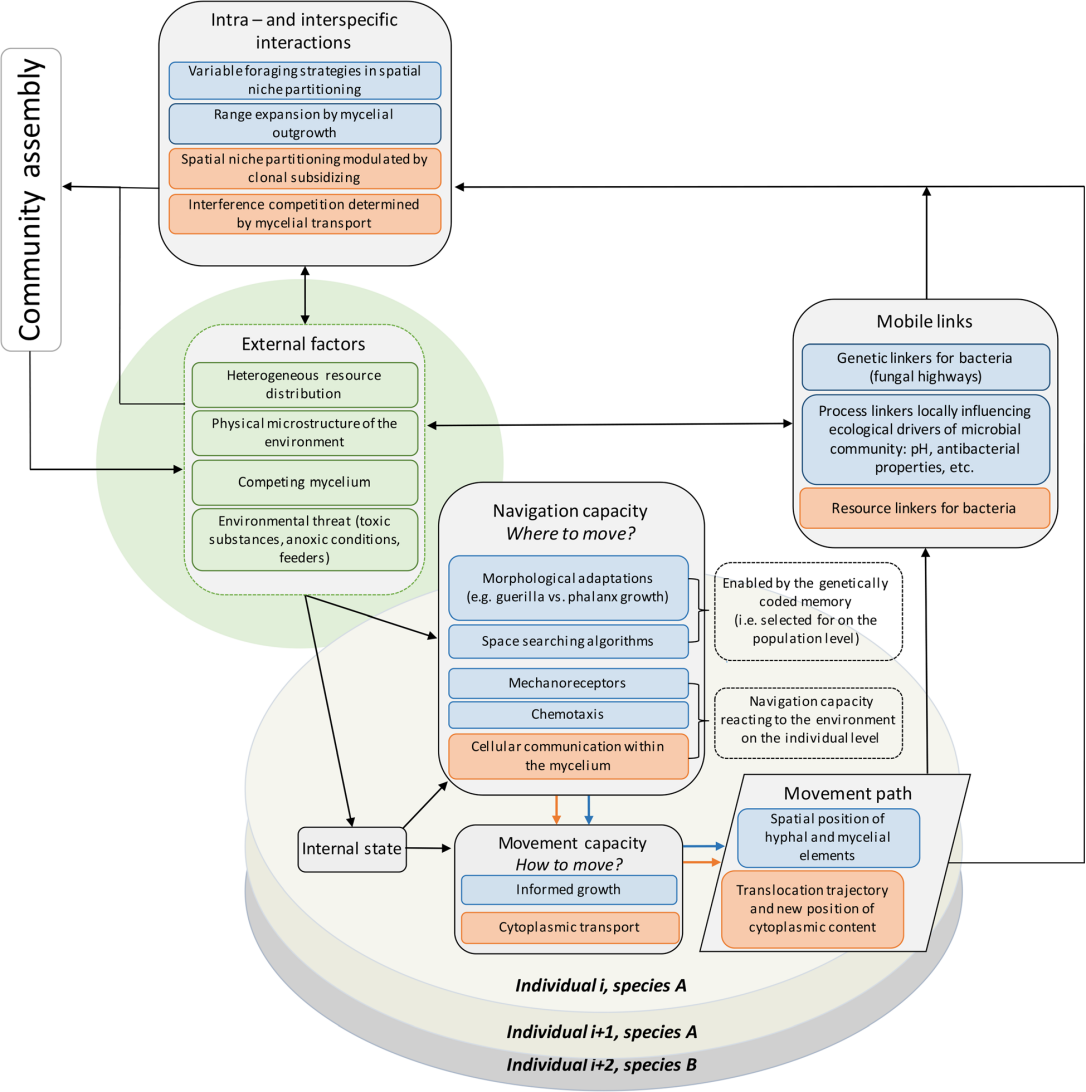
Movement ecology framework adapted for the biology and ecology of filamentous fungi: Original graphical representation of the framework by Jeltsch et al. [4] is combined with fungal movement related phenomena. Blue boxes are related to fungal *active movement* enabled by informed growth. Orange boxes are related to fungal *active movement* enabled by cytoplasmic transport

Both, the growth of hyphae and transport of biomass within the mycelium can be *informed*, i.e. they react to environmental stimuli in order to facilitate interaction of the organism with the environment [16, 17]. In terms of movement ecology, fungi clearly have navigation capacity (Fig. 2). Remarkable navigation capacities are known for example in the grass pathogen *Claviceps purpurea* (the ergot fungus), in which the hypha must pass through several different tissues in order to find its way from the spore germination site to the young floret which it targets [18].

The environmental factors which inform the navigation response (*“Where and when to move?”)*, and thus alter the movement of hyphae and other active biomass translocations (*movement path*) include:

(i) the availability and distribution of resources [17, 19, 20]. The species specific variability in the morphology of the mycelium, for example “phalanx vs. guerilla” foraging strategy is an example of navigation capacity possibly enabled by the genetically coded memory (i.e. potentially selected for on the population level) [19].
(ii) presence of danger in the form of toxic substances or grazers [21, 22]. When fungal mycelium is grazed, the interplay between the internal state (motivation to move and the physiological ability to move), and the navigation capacity can be complex: While intense grazing results (unsurprisingly) in decreased growth, moderate grazing can trigger reactions which can be either interpreted as compensatory growth, or escape mechanisms. Hedlund et al. [22] showed how the fungus *Mortierella isabellina* switches from the normal morphology to the faster growth and increased production of aerial hyphae, in response to grazing by collembola. Authors suggest that aerial hyphae which grow in 3D space have a higher chance to escape the grazer within the pores of a natural substrate. In the Fomina et al. study [21], four different fungal species grew away from the localized sources of Cu and Cd. Interestingly, the response (negative chemotropism) decreased with enhanced availability of sucrose in the medium. Translated to movement ecology terms, this study investigated navigation capacity also in the context of the internal state (i.e. improved physiological options for growing into areas with toxic metals, if enough energy is available).
(iii) presence of conspecific hyphae and mycelia. Hyphae of filamentous fungi are able to use chemotaxis to navigate growth towards other hyphae of the same species, for example between mating partners [23]. Wood decomposing species were also shown to distinguish between different species of competitors, and change their growth between patches accordingly [24].
(iv) the physical structure and other physicochemical characteristics of the environment [25–27]. For instance, in the Hanson et al. study [26], the hyphae were observed within the microstructured environment: Once the fungus grown on an open agar surface enters the microscopic maze, it is able to detect this change, and several growth (movement) parameters are altered. For example, the frequency of branching was increased. This study is also a good example of directional memory in fungi, and its role in navigation. Perera et al. [27] gives an example of how hyphae of dermatophytic fungi use contact-sensing in their navigation through the structures of the host tissues.
(v) the presence of a suitable host (in case of a parasitic or mutualistic fungi). For example, plant roots are known for releasing chemoattractants, which the hyphae use for navigation [20].

### Step 2: active movement in filamentous fungi; Jeltsch’s movement ecology framework

The extended movement ecology concept predicts that not only does the environment have an effect on the *movement path* (see examples mentioned above), but also the interactions that take place along the *movement path* have an effect on the environment, influencing community assembly [4] (Fig. 2). Just like in other groups, in filamentous fungi the focal individual (with its particular movement path) can act as a mobile link (see below) for populations of other guilds. At the same time and within the same guild, movement of this individual is an important factor of fungal community assembly affecting intraspecific and interspecific interactions. Below we expand on these two effects of *active fungal movement*.

#### Hyphae as mobile linkers

The effect of nutrient transport by fungi (i.e. resource mobile links) has been intensively studied in the context of fungus – plant mutualism [28], but the fungi also act as resource mobile linkers for the members of microbial ecosystems. In analogy to the migration of salmon and feeding habits of bears, which result in the creation of nutrient mobile links [29], also the nutrients transported by hyphae can be accessed and released by mycophagous bacteria [30]. However, the nutrient links can have a less dramatic form, where the fungus is not destroyed: In nutrient poor and dry microhabitats, populations of bacteria can be maintained by hyphal transport and excretion of nutrients and water [31]. Mycelia are also able to transport organic contaminants, making them available for biodegradation by soil bacteria [32].

Hyphae of filamentous fungi also act as genetic mobile linkers for populations of soil bacteria [33–35], by providing a network of pathways, which bacterial species can use for their dispersal. In soil, bacteria can typically only move in the water phase. In dry conditions, this can decrease the habitat connectivity. However, connectivity can be improved again by the presence of hyphae surrounded by a water film [34]. The dispersal ability of bacteria on fungal hyphae appears to be a result of a complicated interplay between the traits of both partners. Different fungus - bacteria species combinations show different dispersal potential [36, 37], and the effect has been already shown to influence bacterial community composition [37]. Hydrophobicity decreases the dispersal potential of the fungus [36, 38]. On the bacterial side, the ability to actively move within the water film is important [34], although evidence for passive dispersal also exists [36]. The example of *fungal highways* and *fungal pipelines* (the terms often used in fungal biology for genetic and resource mobile linkers, respectively) also demonstrates how the adoption of general movement ecology concepts in fungal ecology needs to take into account the specifics of microbial communities. For example, since in bacteria the dispersal propagules are usually metabolically active cells, often the function of genetic and resource mobile linker is closely related. As shown above, the fungus not only serves as a passive scaffold, but the dispersal can be further facilitated by provision of nutrients. Dispersal can be also accompanied by the function of *process linkers*. These can be localized pH alterations [39], or *antibiosis*: By creating microenvironments with antibacterial properties, fungi can preferentially spread antibiotic resistant strains [40]. An interesting example is the movement based mutualism between the filamentous fungus *Aspergillus fumigatus* and the swarming bacterium *Paenibacillus vortex* in soil*.* The conidia of the fungus, unable to actively move, can be transported by bacterial populations for distances of at least 30 cm, including from places that do not support fungal growth, into the niches of *A. fumigatus*. In return, the hyphae of the fungal partner serve as bridges for *P. vortex* across soil pore air gaps, which *P. vortex* would be not able to cross on its own [41].

#### Role of active movement in intraspecific and interspecific interactions of fungal communities

Our knowledge of community interactions and assembly in filamentous fungi is still limited, despite the recent advances in this field [1, 42–44]. We argue that this research will benefit from explicit recognition of fungal *active movement* within its ecological context. Below we revisit topics related to fungal intraspecific and interspecific interactions through the lens of movement ecology. Namely mycelial outgrowth as a form of dispersal, mycelial and hyphal foraging, interference competition, and mycelial translocation in clonal subsidizing.

##### Growth and dispersal

Filamentous fungi can regenerate from small hyphal fragments. This means that any type of growth brings also a potential for dispersal. However, this type of dispersal has rarely been addressed in the context of movement ecology, which we think is a missed opportunity. For instance, since colonization is not only restricted to production and release of spores, then addressing the fundamental movement ecology questions of why the fungus grows in exploration mode (e.g. active avoidance of competitors, search for different resources), how is it able to do it (e.g. changing mycelial architecture or growth rate) and when and where to explore (e.g. what cues determine hyphal direction) would better reflect its colonization ability, and may give us a more detailed insight into traditional mycological topics, such as genet mapping [45, 46]. An example of this approach (i.e. how navigation capacity of a foraging fungal individual affects the dispersal on the population level) has been already embodied by Boddy et al. [47]. Authors decided to tackle dispersal by mycelial outgrowth and the resource capture (foraging) as almost synonymous terms. We imagine that this kind of terminology may leave most of the animal ecologists surprised. However, it follows closely and correctly the biology and movement ecology of filamentous fungi.

##### Foraging strategies and niche partitioning

Fungal ecologists have long recognized the existence of different foraging strategies while a fungus explores resources. These include the creation of mycelial cords dedicated to foraging [19], ability to cross an obstacle, or decisions to forage in areas with diverging resource supply [10]. Agerer [48] describes up to eight foraging strategies that root mutualistic fungi exhibit. Boddy and Jones [19] pointed to the morphological variability in fungal species, which can be identified as ‘phalanx or guerrilla’ foraging strategy. Studies of hyphal movements at the microscopic scale also show that foraging strategies (*space searching algorithms*) differ between species [49].

Using the movement ecology framework leads to discussing these findings in terms of coexistence. For example, if species differ in foraging related traits such as the effectiveness of exploring different geometry, then this can lead to spatial niche partitioning.

##### Active movement and fungal interference competition

Interference competition (also known as *fungal combat*) is a well-documented factor of fungal community assembly. We believe the movement ecology framework can offer a new perspective here, since *active movement* plays an important role in two ways: in preemptive competition and in mycelial transport.

Preemptive competition has been identified as one of the main drivers of fungal interference competition [50]. It is known in fungal ecology that the larger the territory of the mycelium at the moment of contact with the competitor, the higher the likelihood of winning the combat [50–54]. The ability of preempting the available space (i.e. primary resource capture) is given by the growth rate of the mycelium. Just like with the directed hyphal growth, this type of growth can also be seen as active movement: Because the incentive to translocate biomass across space is not only developmental (growth), but also ecological (to capture territory and nutrients). Hence, it is not only an analogy to animal growth (increasing the biomass), but also to the animal increasing its fitness by gaining and keeping a territory with its resources (biomass translocation). Besides, preemptive growth is also influenced by the navigation capacity of the fungus, and it can be regarded as a trait important in interspecific variability.

In order for the home range advantage (territory size) to work, the mycelium must not only occupy the resources. Interference competition is resource costly and in an environment with patchy resource distribution the outcomes can depend also on the differences in the ability of the fungus to effectively integrate resources via mycelial transport [17]. Perhaps because this is an obvious conclusion to make, and because of technical difficulties to measure [55], mycelial ability to transport has been - to our knowledge - not explicitly taken into account (i.e. not quantified) in fungal combat experiments (but see Lindahl et al. [53]). Rather, the size of the territory is usually measured, and the influence of transport on mycelial combat outcome is black-boxed, together with other species traits, such as the ability to produce particular biochemical agents, or morphological fortifications. For example, a recent study by Kolesidis et al. [50], which we believe is state of the art in fungal interference competition studies, identified six parameters which can predict the combat outcome. Among them the mycelial extension rate and relative size of the combating mycelia (see above: preemptive competition). The ability to translocate resources is involved in these parameters, and the model parameterized without disentangling it as a separate parameter, can still predict the competition outcome. However - as the authors also discuss - this may not be the case in other instances, for example in a natural environment with patchy resource distribution. Indeed, the experiment was done using a homogenous agar medium. In nature, where resources are patchily distributed across larger spatial scales, interspecific variability in transport capacity can play a major role. The existence of this kind of variability has been already shown in different contexts than interference competition [15].

In the context of interference competition, the importance of transport ability, although not assessed as a trait value across species, was directly or indirectly shown in several studies. When extra resource was made available to *Hypholoma fasciculare* and *Phanerochaete velutina*, there was no difference in the combative ability related to the position of this resource (which was either distal or proximal to the combat zone) [51]. This suggests that the resources from the distal part were readily available in the interference zone. In another study, the resource bases of two species (one saprobic and one mycorrhizal) were separated by a column of soil. Still, the size of the resource base determined the outcome of the interference interaction. The size of the resource also determined the morphology at the interference zone. This is an explicit example of the involvement of mycelial translocations in the outcome of interference competition [53]. Transport can also be hypothesized as one of the reasons behind the observation that spatial configuration of mycelia influences the combat outcome, irrespectively of the mycelia size [56].

Given our knowledge about the importance of mycelial transport in interference competition, and our knowledge about the variability in transport abilities from different research contexts, we can envision studies which track the impact of mycelial transport on interference competition in a more explicit way: With mycelial translocations being quantified as a movement trait value across various species. And with the mycelial translocations seen as *active movement* phenomena, where questions like when and where to move are central. Hence, in a way similar to how movement ecologists look at the relationship between movement and competition.

### Nutrient translocation in a heterogeneous environment, and clonal subsidizing

As described above, filamentous fungi can use mycelial transport to integrate resources from different patches, but this movement ability (trait) differs among species [15]. It is therefore possible that species coexistence can be promoted alongside the trade-off between the ability of resource integration and faster growth. Similarly, species may differ in the ability to transport metabolites into the parts of mycelium, where growth is temporarily not possible due to locally adverse environmental conditions (for possible trade-off in fungal network cost and transport efficiency, see Heaton et al. [55]). The ability to transport nutrients and metabolites across the entire mycelium (i.e. genet) in order to support local parts (ramets) is a feature not unique to filamentous fungi. It connects them for example to clonal plants, where the impact of this form of *active movement* has been already studied within the coexistence context (see below and [57]).

## Interdisciplinary opportunities between movement ecology and fungal biology

There are several ways in which we expect our concept to facilitate interdisciplinary collaboration among movement ecologists and fungal biologist, which would be beneficial for the development of both fields. Here, we expand on several specific examples.

### Use of common language and concepts to assist data and theory synthesis

Our concept can improve the transfer of knowledge from fungal ecology to movement ecology. As shown above, the concept helps translate the relevant knowledge in fungal ecology and biology into a form accessible for movement ecologists. We argue that this is needed given the research gaps in movement ecology; Holyoak et al. [58] identified the problem of inconsistent movement terminology among different taxonomic groups and disciplines. Moreover, they admit that their review was probably biased against microorganismal movement. This happened, because during the screening for relevant articles, they had to exclude several keywords often used in the microorganismal movement research (e.g. *chemotaxis*). These keywords proved to be impractical, as using them identified a large number of articles relevant rather for the fields of molecular biology and cell biology. In fungal biology, movement phenomena are often described using specific terminology. For example, the results of the studies about fungal *space searching algorithms* are highly relevant for (fungal) *foraging* topics, however the term *foraging* was not used in the articles which we reviewed [26, 49, 59, 60]. Had these researchers discussed their findings within the context of foraging, this work would have reached a broader audience and facilitated knowledge transfer. Similarly, we hope that our concept will inspire a debate among fungal ecologists to discuss fungal nutrient translocation also as a form of *nutrient mobile links*, and to complement the term *fungal highways* by the established ecology term *genetic mobile links*.

The concept of *active movement* based on movement ecology can also have a unifying function within the field of fungal ecology and biology. There are now several research groups that work on topics (potentially) related to the *active movement* of fungi and its relevance for microbial ecosystems. Lynne Boddy pioneered the use of established ecological terminology (e.g. *foraging*) advocated above, but she also went further and conceptualized several *active movement* related phenomena as an important factor in the ecology of wood decomposers (for an interesting example, see [19]). Further, clear links between movement and community assembly are now made by the researchers of *fungal highways,* i. e. fungi as *genetic mobile links* for bacteria, and we already pointed out the potential of the *space searching algorithms* research (see: above). We argue that these and other research lines could acquire an added value, if the results were made comparable by discussing them within our unifying framework.

Finally, movement ecology can develop truly universal concepts and terminology only if it includes all ecologically relevant movement phenomena in all groups of organisms. This is a general aim of the discipline [3]. Yet we argue that focusing on animals and propagules of plants leads to sometimes missing this general goal in particular instances. For example, movement ecologists describe the object of their studies as movement of *whole organisms* and *propagules* [3, 58]. The adjective *whole* is used to exclude movement types not directly relevant from the ecological perspective, e.g. the movement of appendices, or physiological movements. As we have shown above, this is perhaps too restrictive. Explicitly recognizing the ecologically relevant *active movement* in diverse groups can improve this terminology (see above: Active movement: definition; or Fig. 1 and Fig. 2).

### Movement ecology, fungal ecology, and communities of plants

Most movement ecology research is focused on motile unitary animals. For this kind of research, unitary motile microorganisms (bacteria, yeast, protists) will be probably a better model system than filamentous fungi. Fungal movement and interactions with the environment are probably too different to serve as a useful model for studying the ecology and evolution of motile unitary organisms. For the same reason however, the overarching framework provided by movement ecology can be very useful in bringing closer together the research on ecology and evolution of clonal plants and filamentous fungi.

As shown by Boddy and Jones [19], there are clear analogies between the *active movement* (growth) of fungal mycelium and clonal plants. Using common terminology and concepts can facilitate integrating theoretical knowledge from clonal plants research into fungal ecology. Conversely, filamentous fungi with their short generation time, feasibility of laboratory cultivation and accessible genome can prove to be useful models to advance research on movement ecology and evolution of clonal metazoa. Evolutionary experiments in fungi similar to those in plants, with selection pressure on phalanx vs. guerilla foraging could be designed [61]. With our current state of knowledge, it is also easy to imagine how fungal ecology could benefit from answering classical questions in clonal plants ecology, as summarized for example by Callaghan et al. [62], or Liu at al [63].: As we already mentioned above (in the context of genet mapping), both fungal and plant ecology are interested in questions around what is the relative contribution of clonal spread vs. dispersal by sexual spores in natural populations, e.g. what is the relative contribution of different dispersal modes in different species and environmental conditions [45, 46, 64, 65]? How does transport of nutrients work in order to increase the likelihood of genet survival? To what extent do ramets collaborate as part of one individual, to what extent do they compete as interconnected individuals, and what is the role of directed growth and transport in this? Further, can clonal integration in fungi decrease species richness by bypassing the niche partitioning options otherwise provided by environmental heterogeneity [57]? Similar to the situation in fungi, in plant ecology there is also limited knowledge about how clonal integration and related movement traits are translated to the community level [63]. Experiments with communities of filamentous fungi are less demanding in terms of both space and time, while assaying the analogous experiments in clonal plants can be more straightforward. Hence, experiments on clonal plants and fungi can complement each other. Model organisms would need to be selected taking into account traits related to clonal *active movement* and environmental interactions. For this, our unifying concept and terminology based on movement ecology framework will be useful.

### Navigation and motion capacity in fungi and slime molds

Our movement ecology based perspective on *active movement* can be applied to all modular organisms. In addition to clonal plants, another notable example is the case of slime molds.

In plasmodial slime molds, navigation and movement capacity has been studied extensively (although not termed this way). What is interesting from the comparative perspective is that these two groups of organisms, however phylogenetically distinct, combine remarkable similarities with important differences.

Both have a clonal and undetermined body plan with hierarchical, transitory biological individuality: The original individual can be separated into several, independently moving individuals, while, unlike in clonal plants, these newly formed individuals can later merge again [66]. In terms of movement ecology framework, intraspecific competition (i.e. isogenic individuals are expected to compete for exactly the same niche space) can be swiftly converted into cooperation, upon the merging of two isogenic individuals into one (Fig. 2).

In both groups, navigation capacity occurs without any neural system, or any other processing center. Both filamentous fungi and plasmodial slime molds intertwine the developmental growth function with the foraging movement function (Fig. 1) [67]. Foraging is realized either exclusively (fungi) or optionally (slime mold) by the informed growth of the reticulated network of tubes, and it is likely that there are similarities in the mechanism of signal propagation, critical for navigation capacity [68].

In both, the physiological (i.e. homeostasis) function of body fluids (cytoplasmic content) is intertwined with the ecological function of integrating different resource patches (Fig. 1) [67]. The network of the mycelium and plasmodium can be remodeled in order to interconnect the resource patches in an efficient way [9, 47, 69, 70].

While most studies about *Physarum polycephalum* did not address the community level, there is one notable exception. Reid et al. [71] studied how slime molds respond to the extracellular secretion (used to mark already explored patches). Its navigation capacity can inform the organism not only about the presence of secretion, but it is also able to distinguish between conspecific and heterospecific secretion. If no fresh patch is available, the slime mold will move into the patch with the heterospecific secretion (after an individual of the same species, there will be fewer resources left). This study clearly demonstrates that not only in animals, movement acts as a factor in community interactions, which can be fully described using the movement ecology framework sensu Jeltsch [4]. Another important lesson from this and similar studies of *Physarum polycephalum* is that even in microbes, the navigation capacity and its effects on community level is not limited to simple responses to environmental cues (e.g. positive or negative chemotaxis), but involves higher order processing, analogous to “decision making” in animals (see also [67, 72]). In fact, indications exist that filamentous fungi are also able to “make decisions”, which in turn can affect community composition. Boddy and Abdalla showed how mycelia can preferentially colonize (discriminate between) resource patches of different quality in terms of presence/absence of a competitor, or even discriminate between competitors of different species [24]. In a study by Holmer and Stendil [52], the cord-forming species *Resinicium bicolor* changed the direction of cords depending on how many resources were available for the combat. In cases when the replacement of the competing *Heterobasidion annosum* was possible, *R.bicolor* oriented its cords towards the competitor. In cases with lower resource availability, *R.bicolor* oriented its cords away from the competitor. It would be interesting to study how the movement related “decision making” of fungi differs between different species, and how this trait affects the community composition.

However, there are important differences between the movement-like phenomena in slime molds and filamentous fungi. First, in fungi, individual hyphae (i.e. a filament) explore microscopic soil structures while forming a potentially macroscopic mycelium. In contrast, in slime molds, the soil microstructures are usually explored by the microscopic (single nucleus, non-plasmodial) amoeba or amoeboflagellate, which is an independent life cycle stage [73]. Second, it is true that some fungi are able to recycle their biomass, which in effect can lead to the translocation of entire organisms. However, this always depends on informed growth on one site of the mycelium, and degradation on another. In contrast, plasmodial slime molds are able to use amoebal movement to translocate their entire body, while this movement does not need to be intertwined with the (informed) growth and biomass recycling. In other words, they are able to translocate also in a more “classical”, animal-like way [73].

And finally, while both slime molds and fungi need to avoid foraging in already explored patches, the mechanisms which they apply are rather different. Slime molds use an extracellular secretion (external memory) to mark already explored patches [71]. In contrast, fungi use hyphal space searching algorithms [59].

## Conclusion

We reviewed studies in fungal biology and ecology through the lens of movement ecology, and proposed an inclusive definition of *active movement*; our definition covers all movement types which organisms from diverse groups can employ in order to interact with their environment. In the case of filamentous fungi, these movement types are informed growth and morphology, directed translocation of substances within the mycelial network, and translocation of entire cytoplasmic contents within hyphae.

Although studies on various forms of biomass translocation in filamentous fungi are rarely framed in a community ecology context, the *active movement* of fungi is likely important for fungal (microbial) community dynamics. Active movement abilities are variable across species and at the same time crucial for the response of the fungus to environmental challenges. That is, they can be viewed as an important fungal movement trait. We showed that fungal studies with different research aims, using diverse techniques, studying diverse scales of organization, movement phenomena, and fungal species, can be all organized under the same umbrella of movement ecology. We think that formalizing what represents ecologically significant movement in fungi can jump start interdisciplinary collaboration between movement ecology and ecology of fungi and other modular organisms. Movement ecology can more efficiently tap into the data gathered by fungal research, and improve the universality of its terminology and framework. Fungal ecology can benefit from the theoretical developments in the field of movement ecology.

We have now rapidly developing technical options for studying fungal network properties, translocations, and hyphal growth on the one hand. On the other hand, movement ecology provides the theoretical background and terminology for thinking about fungal translocations as movement traits important in intraspecific and interspecific interactions. Armed with theory, technical tools, and knowledge from previous fungal ecology research, we can now study fungal translocations with the aim of improving our understanding of fungal ecology and community development.

Specially in soil microbial habitats, the concept proposed here can help answer the recent call by several authors to further pursue the research of microbial communities at the microscale, while taking into account the traits related to this highly heterogeneous and complex environment [74–77].

In addition to this, language of movement ecology in its more universal form, as proposed here, can be used as a basis for the use of filamentous fungi as models in clonal plant ecology, or for comparative studies between filamentous fungi and slime molds.

## Data Availability

Data sharing is not applicable to this article as no datasets were generated or analysed during the current study.
